# Effect of High-Energy Electron Beam Irradiation on the Structure and Thermoelectric Properties of Polypyrrole

**DOI:** 10.3390/polym16243572

**Published:** 2024-12-21

**Authors:** Jie Shang, Jia He, Ziheng Xu, Yufeng Zeng, Yihan Wang, Kun Zhang

**Affiliations:** 1 Shanxi Key Laboratory for Radiation Safety and Protection, CNNC Key Laboratory for Radiation Protection Technology, China Institute for Radiation Proctection, Taiyuan 030006, China; shangjie861220@163.com; 2 Key Laboratory of Radiation Physics and Technology, Ministry of Education, Institute of Nuclear Science and Technology, Sichuan University, Chengdu 610064, Chinakzhang@scu.edu.cn (K.Z.); 3 Sichuan Environment and Protection Engineering Co., Ltd., China National Nuclear Corporation, Guangyuan 628000, China

**Keywords:** thermoelectric materials, conducting polymer, electron beam irradiation, polypyrrole, radiation resistance

## Abstract

The effects of different doses (10–100 kGy) of electron beams on the molecular structure, microstructure, and thermoelectric properties of polypyrrole (PPy) under high-energy electron beam irradiation (10 MeV) were studied. The results showed that after electron beam irradiation, the conductivity of PPy increased slightly, but the Seebeck coefficient and power factor remained relatively stable. The structural analysis of FTIR, Raman spectroscopy, and X-ray diffraction indicated that the molecular structure of PPy was strongly stable, and its microstructure was only slightly affected by electron beam irradiation. Within different dose ranges, the particle size of PPy remained unchanged, indicating that PPy has outstanding radiation resistance performance.

## 1. Introduction

Thermoelectric materials can directly convert heat into electricity. The devices prepared with thermoelectric materials have the advantages of light weight, no noise, environmentally friendly, long service life, etc., and have broad application prospects in the fields of thermoelectric power generation, energized refrigeration, and radioisotope batteries [1]. The efficiency of the thermoelectric materials is generally measured using the dimensionless ZT value [2]: $ZT=S^{2}\frac{T}{\rho \kappa }=S^{2}\frac{\sigma T}{\kappa }=PF\frac{T}{\kappa }$. S indicates the Seebeck coefficient (μV K^−1^), T (K) is the temperature difference between the high temperature side and the low temperature side, σ (S cm^−1^) is the conductivity, and κ (Wm^−1^ K^−1^) is the thermal conductivity of the material. Compared to inorganic thermoelectric materials, polymer-based thermoelectric materials have the advantages of easily adjustable conductivity, light weight, good flexibility, and easy processing and molding, which has attracted more and more attention in the research field of flexible thermoelectric devices. Treatments with charged particle beams, such as low-energy ion beams, X-ray photons, electron beams, γ-rays, protons, and neutrons, provide powerful tools to modify the physical, chemical, thermal, structural, dielectric, and morphological properties of polymers. Among these irradiation techniques, electron beam irradiation is universally applicable, which can be carried out in the final state of samples at normal atmospheric conditions without inducting impurity. When an energetic electron beam is impinged on polymer materials, the dissipated energy can cause degradation, unsaturation, cross-linking, and oxidation thus leading to molecular structure modification and variation. Komilovh and Lachinov [3] found that electron beam irradiation resulted in a decrease in the band gap of the poly (phthalamide) diene (PPB) polymer films due to the ring opening reaction of the lactone structure. C Ranganathaiah et al. [4] studied the effects of an electron beam with energy of 8 MeV on the microstructure and electrical properties of bakelite. The results showed that when the electron beam dose was at 20–40 kGy, OH^−^ and H^+^ radicals were produced by chemical bond breakage in the bakelite molecular chain, leading to an increase in the volume of positively charged radicals in the amorphous region and an enhancement in conductivity. When the dose exceeded 40 kGy, the previously generated free radicals underwent rearrangement and chemical cross-linking, which hindered the charge transfer between molecular chains and resulted in a decrease in conductivity. Chaudhary et al. [5] found a similar trend in the study of polyaniline silver (PANi/Ag) composite films. When the dose of the electron beam (10 MeV) was less than 30 kGy, the conductivity was enhanced due to the doping effect of the free radicals generated by PANi molecular chain breakage; when the dose exceeded 30 kGy, conductivity was reduced because of the cross-linking of free radicals. Therefore, the previous studies suggest that electron beam (EB) irradiation can effectively tailor the molecular structures and charge transport features of polymer materials. With the development of various applications of functional polymers in the electronuclear industry, such as radiation monitoring [6], radioisotope thermoelectric generators, heat pipe reactors [7], and thermoregulatory coatings for space exploration [8], research about the irradiation stability of conductive polymers is highly desired. Polypyrrole (PPy), as a common intrinsically conductive polymer, was considered as a promising candidate for TE materials [9]. PPy can be doped with dopants such as cetyltrimethylammonium bromide (CTAB), p-toluenesulfonic acid (TSA), ferric chloride (FeCl_3_), and sodium dodecyl sulfate (SDS) to increase thermoelectric properties [10]. The electrical conductivity of the doped state of PPy is closely related to the structure of the N atoms, such as the presence of uni-polarons and bi-polarons, the conjugate size of the pyrrole ring, and the formation of hydrogen bonding between the N atoms [11]. One of the biggest advantages of PPy is that the structure of PPy is highly stable, and it remains to be studied whether electron beam irradiation can affect its structure and produce a better modification influence on PPy.

In this work, PPy doped with FeCl_3_ was irradiated under 10 MeV EB with increasing dosage from 10 to 100 kGy. After electron beam irradiation, the conductivity of PPy increased slightly, but the Seebeck coefficient remained relatively constant. The maximum σ and PF value of irradiated PPy was up to 400 S m^−1^ and 0.016 µ W m^−1^ K^−2^, both about 1.8 times that of pristine PPy (221 S m^−1^ and 0.0094 µ W m^−1^ K^−2^, respectively). Structural analyses revealed that little change in molecular chains of PPy was induced by energetic EB irradiation, suggesting that PPy possessed good radiation resistance performance.

## 2. Materials and Methods

### 2.1. Experimental Methods

Raw materials: Pyrrole (CP, purity ≥ 98.0%, Aldrich, Burlington, MA, USA) was purified by vacuum distillation before reaction. Anhydrous ethanol (AR) and FeCl_3_·6H_2_O (AR) were obtained from Chengdu Kelong Chemical Co., Ltd., Chengdu, China. All the reagents were used as received without any further purification except pyrrole. Deionized water was used throughout.

**Figure 1:**
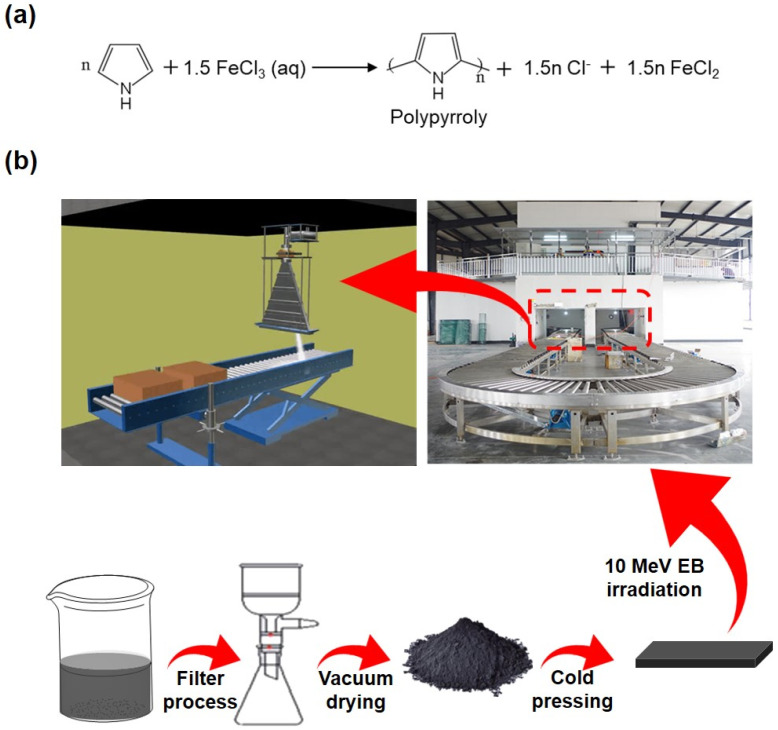
(**a**) Synthesis reaction formula of polypyrrole. (**b**) Process of PPy formation and EB irradiation (red dotted: irradiation zone).

1. PPy preparation: Firstly, PPy doped with FeCl_3_·6H_2_O was prepared. In the typical procedure, pyrrole monomer (1 mL) was dissolved in 300 mL of FeCl_3_·6H_2_O (5.87 g) aqueous solution, and the reaction was carried out under stirring at room temperature for 12 h. After polymerization, the resulting product was filtered and washed with anhydrous ethanol and deionized water in sequence several times until the filtrate was clarified. The final product was dried under vacuum at 60 °C for 24 h and named PPy. The reaction equation is shown in Figure 1a.
2. E-beam irradiation experiments: E-beam irradiation experiments were carried out using a DZ-1020 electron linear accelerator at Suining Guangfa Irradiation Technology Co., Ltd., Suining, China. The beam current was 2 mA with a conveyor speed of 2.6 m/s, and the scanning width was 0.8 m. The final irradiation doses were 10, 20, 30, 40, 50, 60, 70, 80, 90, and 100 kGy, respectively. The process of PPy formation and EB irradiation was illustrated in Figure 1b.

### 2.2. Characterization

Structural characterization: Fourier transform infrared spectroscopy (FT-IR) was obtained using a German BrukerTensor27 infrared spectrometer to study changes in molecular structure. For the powder samples, the KBr pressing method was used and the tablets were pressed under a pressure of 10 Gpa for testing. Differential scanning calorimetry (DSC) was estimated by Switzerland METTLER Toledo DSC1 model with a heating rate of 10 °C min^−1^ under N_2_ atmosphere. A French LabRAM HR laser Raman spectrometer was used, with a test range of 500–2200 cm^−1^ and a laser wavelength of 532 nm. Scanning electron microscope (SEM) was carried out using a Nova Nano SEM 450 scanning electron microscope to observe the microscopic morphology of the samples, with a test voltage of 5 kV, and the test was conducted after spraying gold. A Bruker D8 Advance X-ray diffractometer was used for X-ray diffraction (XRD) measurements, with Cu Kα (λ = 0.1542 nm) as the ray source, the scanning range was 10~80°, and the scanning speed was 12° min^−1^, in which the measurement of PPy powder was performed by grazing incidence measurement with an incidence angle of 1°.

Thermoelectric characterization: The electrical conductivity Seebeck coefficients were measured using (Namicro-3, JouleYacht, Wuhan, China) under vacuum at 300 K. For the powder samples, cold press molding was used, and the samples were pressed into 1 mm × 5 mm × 15 mm rectangles at a pressure of 10 MPa. The resistivity can be obtained by measuring the electromotive force at both ends of the sample with the known current according to the formula *ρ* = *VA/IL* = *RA/L* (*V*: electromotive force between two probes; *I*: current; *L*: distance between two probes; *R*: resistance; *A*: cross-sectional area of the sample perpendicular to the current direction). Finally, the conductivity is calculated by the formula *σ* = *1*/*ρ*. Thermoelectric voltage across the films was measured at ten different temperatures between −10 and +10 K and the Seebeck coefficient was obtained from the slope of the temperature gradient–voltage plot.

Computational methods: Molecular structure of PPy and irradiated PPy molecules were carried out for molecular dynamics simulations to obtain their interactions by Material Studio package [12]. Density functional theory (DFT) calculations were performed to investigate the electronic structure of PPy and irradiated PPy. All configurations of PPy and irradiated PPy were constructed and optimized using the Dmol3 module. For the exchange-correlation functional, the generalized gradient approximation (GGA) was applied within the Perdew–Burke–Ernzerhof (PBE) formulation [13]. The charge distribution and HOMO-LUMO orbitals of PPy and irradiated PPy were studied. 

## 3. Results and Discussion

### 3.1. Thermoelectric Property

**Figure 2:**
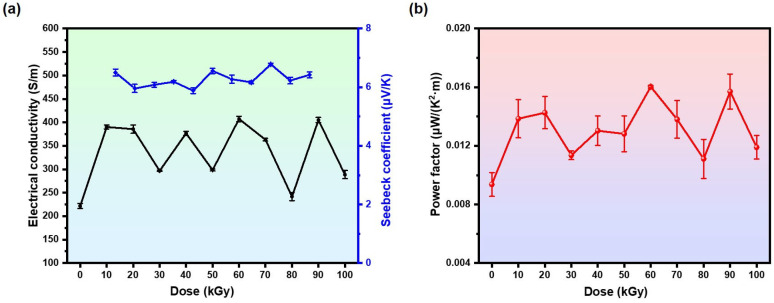
(**a**) Electrical conductivity and Seebeck coefficient, and (**b**) power factor of PPy samples after electron beam irradiation.

Figure 2 shows the thermoelectric properties of PPy samples after electron beam irradiation. As shown in Figure 2a, in the low-dose region of 0–30 kGy, the conductivity of pristine PPy has a global minimum value of 221 S/m and reaches a local maximum value of 390 S/m at 10 kGy. In the medium-dose region of 40–60 kGy, the PPy-50 kGy sample has a local minimal value of 298 S/m and a global maximum value of 407 S/m at 60 kGy. Finally, in the 70–100 kGy high-dose region, the conductivity of PPy has a local minimal value of 241 S/m at 80 kGy and a local maximal value of 404 S/m at 90 kGy. This result suggests that electron beam irradiation has a positive effect on the electrical conductivity of PPy. The Seebeck coefficient fluctuates slightly around 6 μV/K (Figure 2a), with a value of 6.57 μV/K for the pristine PPy, a maximal value of 6.78 μV/K at 80 kGy, and a minimal value of 5.88 μV/K at 40 kGy. It is worth noting that the electrical conductivity showed the opposite trend with the Seebeck coefficient, which agrees with the mathematical relationship between conductivity and the Seebeck coefficient, as described by Equations (1) and (2).

(1) $$S\propto {\left(\frac{\pi }{3n}\right)}^{\frac{2}{3}}$$

(2) $$\sigma =\sigma_{0}\exp\left[-{\left(\frac{T_{0}}{T}\right)}^{\frac{1}{4}}\right]$$

where *n* is the carrier concentration, *T* is the absolute temperature, *T*_0_ is characteristic temperature depend on the localization and density of the states, and σ_0_ is the conductivity under T_0_ [14].

The power factor of PPy after irradiation also showed no obvious trend of changes (Figure 2b), but still had a minimum value of 0.0094 μV/K^2^m at un-irradiated and a maximum value of 0.016 μV/K^2^m at 60 kGy. The above results showed that electron beam irradiation could not significantly affect the thermoelectric properties of PPy, indicating that PPy has strong resistance to irradiation and can still maintain stable thermoelectric properties under high doses of electron beam irradiation. This property makes PPy promising candidates in applications involving radiation environments, such as in space exploration.

### 3.2. Molecular Structure 

**Figure 3:**
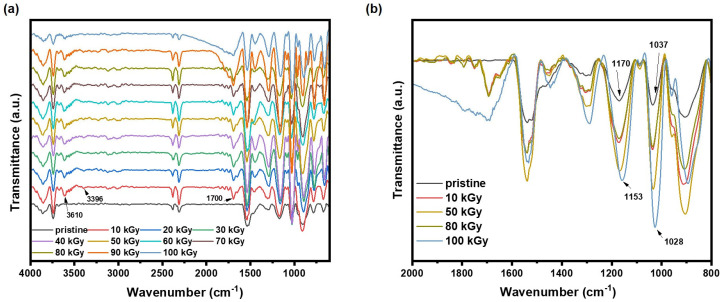
(**a**) Full spectrum of 600–4000 cm^−1^ and (**b**) magnified image of 800–2000 cm^−1^ of FT-IR spectrum of pristine and irradiated PPy.

The infrared spectrum of electron beam irradiated PPy is shown in Figure 3a. The peak at 894 cm^−1^ is generated by the out-of-plane bending vibration of C-H, whereas the peaks at 1037 cm^−1^ and 1286 cm^−1^ correspond to the in-plane bending vibration of C-H, and the peak at 1170 cm^−1^ is attributed to the stretching vibration of C-N. The peaks at 1463 cm^−1^ and 1535 cm^−1^ represent the vibration of the pyrrole ring, and the stretching vibration of C=C [15], respectively. The peaks near 3610 cm^−1^ are attributed to the stretching vibration of the pyrrole cyclic imine [16], and the peak at around 3396 cm^−1^ is the stretching vibration of -OH. Peaks at around 1700 cm^−1^ correspond to unsaturated -C=O. In the enlarged image of 600–2000 cm^−1^ in Figure 3b, when irradiation doses were less than 100 kGy, peaks near 1537 cm^−1^ in all the PPy spectrum changed slightly, but at 100 kGy, the peaks were red-shifted to 1533 cm^−1^ and became narrower, and the peaks at 1170 cm^−1^ and 1037 cm^−1^ were also red-shifted to 1153 cm^−1^ and 1028 cm^−1^, respectively. According to the study by Xu Jinyu et al. [17], the C-N bonds can interact with other polar groups of PPy molecules to form hydrogen bonding interactions, which contributes to the red-shift of the related spectral peaks. Whereas the study by Nanda Kumar Hota et al. [18] showed that electron beam irradiation can indeed achieve molecular cross-linking by enhancing the hydrogen bonding interactions of polymer molecules. This suggests that some of the nitrogen atoms in PPy may have gained electrons at a high electron beam irradiation dose, leading to an increase in the cross-linking of the PPy molecules and changing in the vibrational modes of the neighboring pyrrole ring.

**Figure 4:**
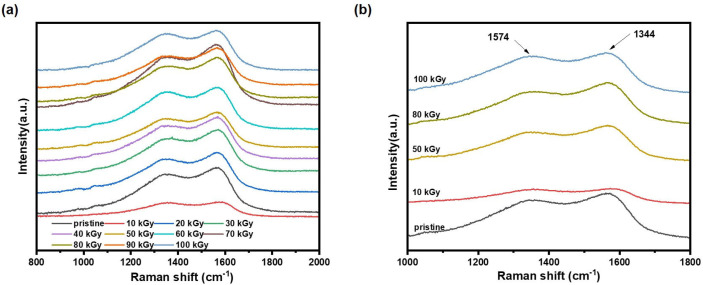
Raman spectra of pristine and irradiated PPy: (**a**) full spectra of all samples; (**b**) partial spectra from 1000 to 1800 cm^−1^ of samples at 0, 10, 50, 80, and 100 kGy.

Since no obvious changes were observed for the samples in the IR spectra, the samples were further investigated by Raman spectroscopy. As shown in Figure 4a, only two characteristic peaks at 1575 and 1384 cm^−1^ were observed for all the samples, which were ascribed as C=C stretching vibrational mode and the C-N anti-symmetric stretching vibrational mode, respectively [19]. This also indicated that the PPy samples were mainly Cα-Cα type polymerization. Apart from this, the peak at 1574 cm^−1^ was blue shifted to near 1586 cm^−1^ (Figure 4b) only in the 10 kGy sample, suggesting that the increase in the degree of pyrrole ring deviation of the sample resulted in the enhanced conductivity of the 10 kGy sample. However, this peak of all the other samples remained at the same position. Based on the above analysis, the overall structure of PPy is very stable at irradiation doses of lower than 100 kGy, while higher irradiation doses lead to increased intermolecular hydrogen bonding.

**Figure 5:**
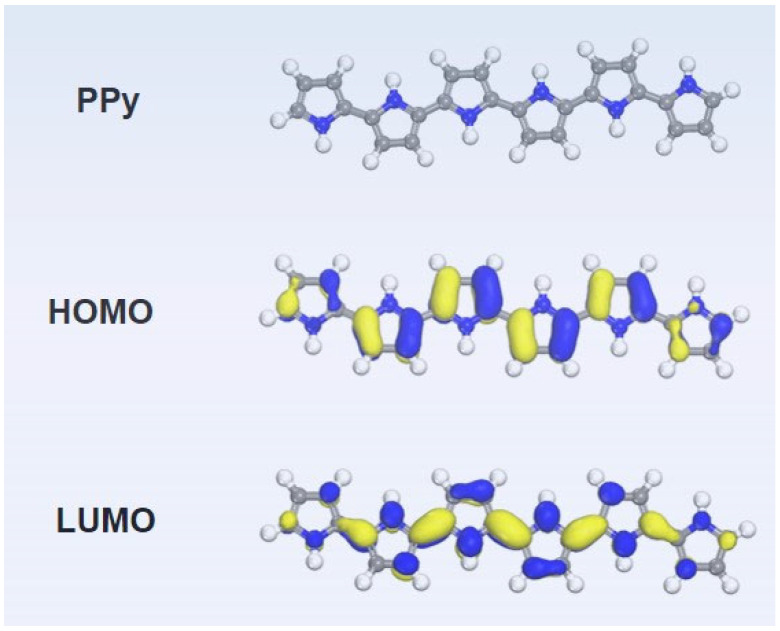
HOMO and LUMO distribution of simulated PPy molecules.

**Figure 6:**
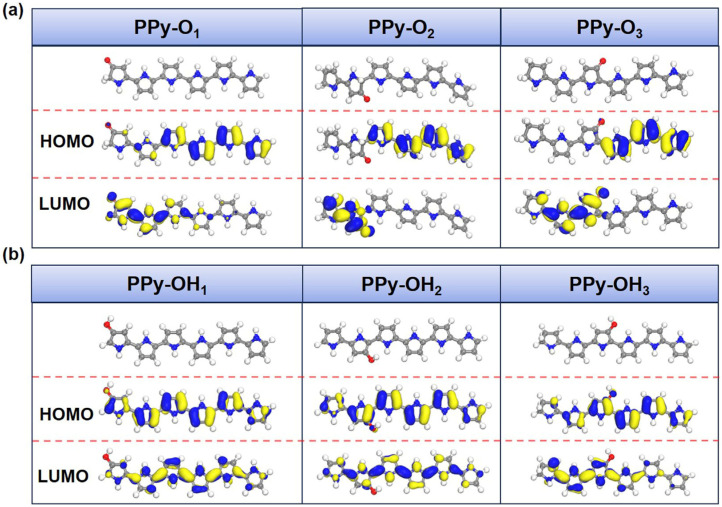
LUMO and HOMO distribution of simulated (**a**) PPy-O and (**b**) PPy-OH molecules.

Based on the aforementioned analyses, the mechanism of the thermoelectric enhancement in EB irradiated PPy is proposed as below. As is known, radiation-induced reactions in polymers are triggered by the rapid distribution of absorbed energy within the molecule, resulting in the generation of free radicals. In PPy, the five membered ring skeleton in PPy possess good irradiation stability because the π-bond structure can disperse radiation energy effectively, making the pyrrole units stable under radiation, which can be recognized in the main FTIR absorption peaks. However, when PPy are subjected to high-energy EB irradiation, radicals will react rapidly with O_2_ to produce peroxy radicals (RO_2_·). These radicals can form active species such as alkoxy radicals (RO) and hydroxy radicals (OH). Then, when all random free radicals recombine and react to form stable molecules (R=O or R-OH), termination reaction occurs [20]. To further study the influences of PPy with the oxygen-containing functional group on the thermoelectric properties of PPy, the DFT calculations were performed to investigate their electronic structure, as shown in Figure 5 and Figure 6.

In Figure 5 and Figure 6, a visual depiction of the LUMO-HOMO states of PPy and irradiated PPy obtained with six pyrrole units after TD-DFT calculations is shown. In this case, PPy-O_1_, PPy-O_2_, and PPy-O_3_ refer to irradiated PPy with C=O group on the 1st, 2nd, and 3rd pyrrole units, PPy-OH_1_, PPy-OH_2_, and PPy-OH_3_ refer to irradiated PPy with -OH group on the 1st, 2nd, and 3rd pyrrole units, respectively. The LUMO and HOMO states differ in shape and symmetry of the PPy samples. As shown in Table 1, the band gap (ELUMO-EHOMO) of pristine PPy is 2.16 eV, giving a direct explanation of its low electrical conductivity. After irradiation, the given products, such as PPy-O_1_ (1.84 eV), PPy-O_2_ (1.68 eV), PPy-O_3_ (1.12 eV), and PPy-OH_3_ (2.11 eV) show band-gap reduction, meaning the irradiation benefits the carrier transport and hence enhance the electrical conductivity of PPy. Accordingly, the ups and downs of electrical conductivity from 10 kGy to 100 kGy may come from the differences in the quantity of -C=O or -OH groups.

### 3.3. Crystalline Structure

**Figure 7:**
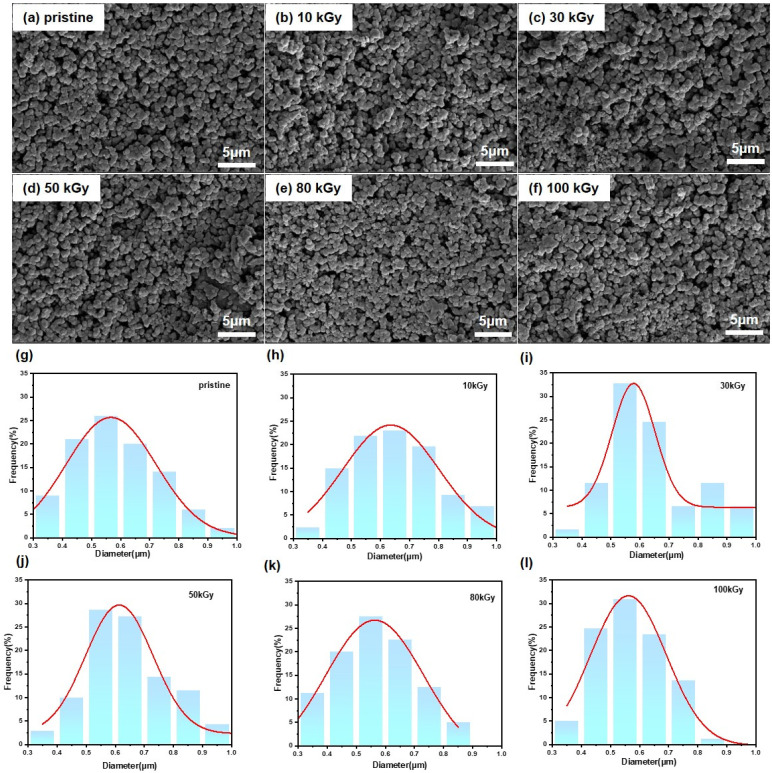
(**a**–**f**) SEM images and (**g**–**l**) particle size distribution of pristine PPy and PPy irradiated at 10, 30, 50, 80 and 100 kGy.

The above results indicate that the molecular structure of PPy changed slightly, suggesting that PPy has good tolerance to electron beam irradiation at the molecular level. In order to further investigate the effect of electron beam irradiation on PPy, its micro-morphology was analyzed. Figure 7 presents the SEM images of pristine PPy and PPy irradiated at 10, 30, 50, 80, and 100 kGy. All the samples clearly showed a tightly arranged spherical structure [21,22], some larger particles were composed of two or more smaller grains, and this structure remained unchanged with the increase in the radiation dose of the sample. In addition, there was no change in the PPy particle size. Therefore, electron beam irradiation has little influence on the degree of arrangement and aggregation of PPy particles.

**Figure 8:**
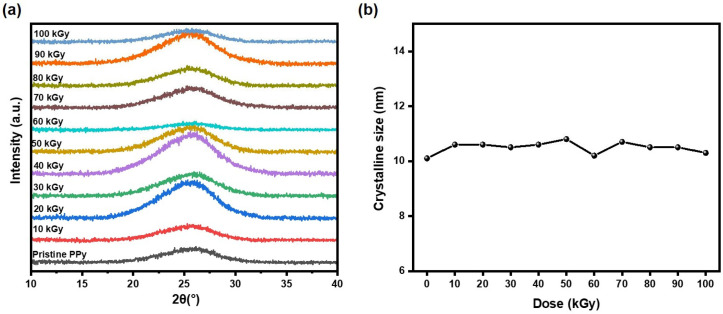
(**a**) XRD spectra and (**b**) crystalline size of pristine and irradiated PPy.

The microstructure of the samples also includes their crystal structure, and XRD is the most direct means to characterize the crystal structure of the samples. The XRD spectra of the electron beam irradiated PPy samples are shown in Figure 8a. All PPy samples exhibit broad peaks, which demonstrate the amorphous nature of PPy [23]. As shown in Figure 8b, the amorphous peak of PPy can be fitted to the position of 25.4°. This position had barely any displacement under electron beam irradiation, meaning it can be concluded that the d-spacing of the sample, that is, the inter-chain distance, stays unchanged significantly. The crystalline size of the sample was calculated according to Scherrer’s formula, as described by Equation (3).

(3) $$D_{sh}=\frac{k\lambda }{\beta cos\theta }$$

k is a constant with a value of 0.89, λ is 1.54 Å, representing the wavelength of the X-rays emitted by the detector; β is the full width at half maximum (FWHM); and θ is the peak position.

In the high irradiation dose range of up to 100 kGy, the crystalline sizes of the samples are also basically the same and are about 10 nm. The calculation results are presented in Table 2, and indicate that the crystal structure of the samples remained unchanged. In order to further verify the stability of the crystal structure, a DSC test was carried out on the irradiated PPy.

**Figure 9:**
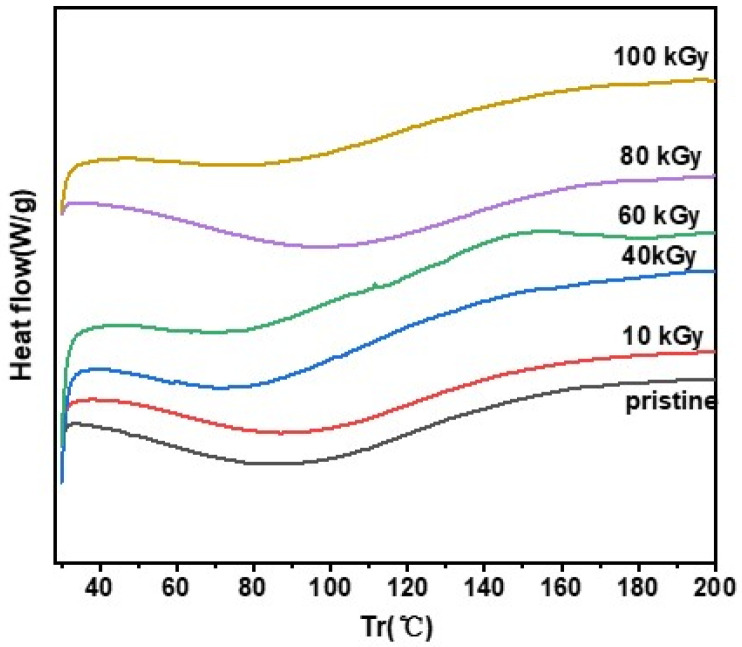
DSC spectra of pristine PPy and PPy irradiated at 10, 40, 60, 80, and 100 kGy.

The result of the DSC test of PPy is shown in Figure 9. There is a clear endothermic depression at about 90 °C, corresponding to the glass transition temperature of PPy. And according to Table 3, this temperature shows a small tendency of increasing first and then decreasing as the irradiation dose of the sample increases. The cutoff temperature of this peak is above 140 °C, which indicates that the sample does not have an obvious glass transformation. During the whole irradiation process, PPy still maintains a high disorder [24], which is consistent with the XRD results.

## 4. Conclusions

The conductive polymer PPy was treated with high-energy electron beam irradiation, and it was found that PPy showed good anti-irradiation performance. Electron beam irradiation only caused small fluctuated changes in the thermoelectric properties of PPy. In terms of molecular structure, the five-element ring structure of PPy was slightly affected, and the overall degree of polymerization was not reduced, while the enhancement of hydrogen bonding was observed at a dose of 100 kGy. In addition, electron-beam irradiation did not damage the arrangement and degree of aggregation of PPy. This research shows that PPy has good anti-irradiation properties against electron beam irradiation and has high potential for application in ionizing radiation environments.

## Figures and Tables

**Table 1:** Energy of simulative PPy and irradiated PPy.

Energy (eV)	Pure PPy	PPy- O_1_	PPy- O_2_	PPy-O_3_	PPy-OH_1_	PPy-OH_2_	PPy-OH_3_
E_HOMO_	−3.70	−4.05	−3.64	−3.72	−3.78	−3.72	−3.65
E_LUMO_	−1.54	−2.22	−2.66	−2.57	−1.52	−1.55	−1.54
E_gap_	2.16	1.84	1.68	1.12	2.26	2.17	2.11

**Table 2:** Crystalline size of samples calculated by Scherrer’s formula.

Irradiation Dose (kGy)	Peak Position (2θ)	FWHM	Crystalline Size of Samples (nm)
pristine	25.78	7.62	10.1
10	25.56	7.34	10.6
20	25.44	7.30	10.6
30	25.70	7.40	10.5
40	25.52	7.30	10.6
50	25.52	7.20	10.8
60	26.00	7.56	10.2
70	25.70	7.22	10.7
80	25.60	7.36	10.5
90	25.50	7.38	10.5
100	25.76	7.52	10.3

**Table 3:** Glass transition temperatures of calculated from DSC spectra of PPy.

Dose (kGy)	0	10	40	60	80	100
Temperature (°C)	90.11	91.95	80.14	76.31	99.75	87.14

## Data Availability

The original contributions presented in this study are included in the article. Further inquiries can be directed to the corresponding author.
